# NOD: a web server to predict New use of Old Drugs to facilitate drug repurposing

**DOI:** 10.1038/s41598-021-92903-8

**Published:** 2021-06-29

**Authors:** Tarun Jairaj Narwani, Narayanaswamy Srinivasan, Sohini Chakraborti

**Affiliations:** Molecular Biophysics Unit, Indian Institute of Science, Bengaluru, Karnataka 560012 India

**Keywords:** Computational biology and bioinformatics, Drug discovery

## Abstract

Computational methods accelerate the drug repurposing pipelines that are a quicker and cost-effective alternative to discovering new molecules. However, there is a paucity of web servers to conduct fast, focussed, and customized investigations for identifying new uses of old drugs. We present the NOD web server, which has the mentioned characteristics. NOD uses a sensitive sequence-guided approach to identify close and distant homologs of a protein of interest. NOD then exploits this evolutionary information to suggest potential compounds from the DrugBank database that can be repurposed against the input protein. NOD also allows expansion of the chemical space of the potential candidates through similarity searches. We have validated the performance of NOD against available experimental and/or clinical reports. In 65.6% of the investigated cases in a control study, NOD is able to identify drugs more effectively than the searches made in DrugBank. NOD is freely-available at http://pauling.mbu.iisc.ac.in/NOD/NOD/.

## Introduction

Drug repurposing strategies aim to identify new uses of existing (old) drugs, thereby aiding the reduction of overall time and costs involved in discovering novel therapeutic agents. Computational approaches play crucial role in accelerating the repurposing ventures by rationally narrowing down the chemical search space, facilitating faster progress to subsequent experimental and clinical investigations. This incredibly helps when : (i) a solution for therapeutic management of a new indication is desirable within a short time, like in the current novel coronavirus pandemic, or (ii) the investments in the research is low, such as in the cases of rare genetic and neglected infectious diseases^1^.

Databases like ChEMBL^2^, DrugBank^3^, DrugRepurposing Online (available at https://drugrepurposing.info/), DrugRepV^4^ hold vast information on known targets, biological activities, and other details of compounds that can guide repurposing endeavours. Effective utilization of such information would often require sophisticated computational skills, and so, web-based automated pipelines are often useful to non-specialist researchers. A majority of the existing web-based drug repurposing tools, such as ProBis-ligand^5^, Drug ReposER^6^, ACID^7^, and eRep-ORP^8^ are dependent on reliable structural information of the target protein. Undoubtedly, structure-based methods provide a wealth of biological information. However, reliable structural data may not always be available. Further, a structure-based approach, like docking as implemented in ACID, has certain limitations^9^: (i) sufficiently high-resolution input structures are required to obtain reliable predictions from docking simulations, (ii) structural flexibility associated with proteins and ligands adds to the uncertainty in the predictions, (iii) limitations in the scoring function can be challenging to select reasonable binding pose/s from among a large number of predicted poses with marginally varying energy values, and (iv) enormous computational resources are required to conduct rigorous large-scale screenings of ligands, which is also time-consuming. Among the other approaches, ligand-guided drug repurposing is offered by SwissSimilarity^10^. There are also several data-driven tools to aid drug repurposing, for example, CMap^11^, Drug Target Profiler (DTP)^12^, and Guldify v2.0^13^. However, the potential of sequence-guided drug repurposing has not yet been extensively explored. A comprehensive list of the mentioned computational drug repurposing resources and their working principles could be found elsewhere^14^.

We have developed NOD, a web server to predict New use of Old Drugs. NOD exploits the evolutionary relationship between proteins to suggest potential repurpose-able compounds that include FDA-approved drugs and investigational drug candidates. Generally, homologous proteins adopt similar structures and may often possess conserved ligand-binding sites^15^. As a result, cross-reactivities of drugs with unintended homologous targets are expected, which is termed polypharmacology^16^. The benefits of polypharmacology mediated by similar binding sites of homologous proteins have been exploited in our earlier sequence and structure-guided computational drug repurposing studies^17–21^. Our predictions are found to corroborate with experimental data. Our learning from the previous studies prompted us to build NOD, which facilitates homology detection between a query and a target protein by a rigorous Hidden Markov Model (HMM)-based iterative sequence search^22^. In many instances, NOD offers an advantage by identifying compounds known to interact with remote homologs which﻿ otherwise remain undetected by simple BLAST searches^23^, as implemented in DrugBank. These compounds could also be potential candidates for repurposing against the protein/s of interest. If NOD detects at least one reliable homolog of the protein of interest, the list of compounds known to interact with the target homolog could be obtained within minutes of the availability of its sequence information, even when the 3-D structure of the protein is unknown. These potential repurpose-able candidates and other relevant details for decision-making are displayed on the web interface of NOD and can also be downloaded as tab-separated files.

## Results

Various studies were conducted to assess the performance of NOD, and the key findings are briefed below.

### Validation

To test the credibility of NOD in being able to make the right Query-Target-Compound (QTC) associations, we ran several jobs with protein targets from different pathogens, such as (a) *Mycobaterium tuberculosis* (Mtb), (b) *Plasmodium falciparum* (P_fal), (c) *Candida albicans* (C_alb), (d) Human Immunodeficiency Virus (HIV), (e) Hepatitis C virus (HCV), and (f) SARS-CoV-2. Besides, selected examples from the two major classes of druggable human targets, i.e., GPCRs and kinases, were also used for the analysis. The selection of all the test cases is guided by their importance in different therapeutic areas as well as influenced by our experiences in computational repurposing of drugs using structure (target or ligand-centric) and/or sequence-based approaches^18–21,24^.

More than 10000 query protein sequences have been tested with the default setting of query coverage (>70%) in NOD. The results include more than 100 compounds that are already known (approved/under-trial) to be interacting with the respective query protein (or its detected homologs) and are proved to be effective in treating the corresponding indication as supported by their laboratory and/or clinical investigation reports. The agreement between the NOD results and established experimental data validates the methodology implemented in NOD (Table 1, Supplementary Table S1).

**Table 1:** Few selected examples from validation of NOD’s predictions.

Sl. No.	Disease (causative pathogen, if applicable)	Query protein (gene)	Target homolog protein (organism, gene)	Potential candidate (DrugBank ID)	Reference to laboratory and/ or clinical evidence
1	Tuberculosis (*M. tuberculosis*)	G0ZF27 (rpoB)	P0A8V2 (*E. coli*, rpoB)	Rifabutin (DB00615)	^25^
2		P9WPL3 (cyp143)	P08684 (Human, CYP3A4)	Isoniazid (DB00951)	^26^
3		P9WH63 (rpsL)	P0A7S3 (*E. coli*, rpsL)	Kanamycin (DB01172)	^27^
4		P9WH63 (rpsL)	P0A7S3 (*E. coli*, rpsL)	Amikacin (DB00479)	^27^
5		G0ZF23 (rpoB)	P0A8V2 (*E. coli*, rpoB)	Rifampicin (DB01045)	^28^
6	Malaria (*P. falciparum*)	Q8ILQ7 (GST)	P09210 (Human, GSTA2)	Chloroquine (DB00608)	^29^
7		Q8I4X0 (PFL2215w)	P63261 (Human, ACTG1)	Artenimol (DB11638)	^30^
8	Candidiasis (*C. albicans*)	A0A1D8PDL5 (GSL1)	A2QLK4 (*A. niger*, fksA)	Anidulafungin (DB00362)	^31^
9		P10613 (ERG11)	P50859 (*C. glabrata*, ERG11)	Isavuconazole (DB11633)	^32^
10		Q5A7M3 (BNA4)	Q14534 (Human, SQLE)	Terbinafine (DB00857)	^33^
11		A0A1D8PJ01 (PMA1)	P05023 (Human, ATP1A1)	Ciclopirox (DB01188)	^34^
12		Q5A7M3 (BNA4)	Q14534 (Human, SQLE)	Naftifine (DB00735)	^35^
13	AIDS (*Human Immunodeficiency Virus*)	Q72874 (pol)	O90777 (*HIV*, HIV-1 protease)	Nelfinavir (DB00220)	^36^
14	Covid-19 (*SARS-CoV-2*)	P0DTD1 (rep)	P0C6X7 (*SARS-CoV*, rep)	Remdesivir (DB14761)	^37^
15	Cancer	O14965 (AURKA)	P24941 (Human, CDK2)	N-[3-(1H-benzimidazol-2-yl)-1H-pyrazol-4-yl]benzamide (DB08066)	^38^

P.S.: In the case where the candidate molecules are associated with more than one query-target pairs, only a single non-trivial (i.e., non-self hit) pair is selected for reporting in this table. Information on detailed validation results of NOD could be found in the Table S1.

### Control study

To the best of our knowledge, DrugBank is the only freely available web-based resource that allows sequence-guided drug repurposing. Therefore, a control dataset comprising 31 query protein sequences from Mtb, P_fal, and C_alb was formed to compare the performance of NOD with DrugBank. Our previous experiences on repurposing drugs against these pathogens guided the selection of the 31 proteins^18,19,21^. In the earlier studies, we could identify remote homologs (with sequence identity ~20%) of these 31 proteins that interact with existing approved drugs, resulting in 32 QTC associations. Only those query-target pairs, for which the target proteins are still available in the current version of the DrugBank database, were selected for the control study. The set of 31 proteins were submitted to DrugBank and NOD. To exploit the power of HMM-based search implemented in NOD to the fullest, we did not impose any query coverage cut-off for the control study.

The control study revealed that for 65.6% (21 out of 32) of the investigated cases, NOD out-performs DrugBank, i.e., NOD offers better coverage of associations than DrugBank in sequence and chemical space. However, for none of the studied cases, DrugBank could out-perform NOD. This implies that while all the associations suggested by DrugBank are also identified by NOD, the reverse is not entirely true. Interestingly, some of the drugs suggested exclusively by NOD have already been experimentally probed for their effectiveness against the related diseases (Table S2). Examples include predicting the repurposing potential of: (i) Triclosan and Cefatolin in treating tuberculosis, (ii) Triclosan and Mupirocin in treating malaria. Nonetheless, many suggested associations are new findings and demand further investigations (Table S2). These examples include prediction of repurposing potential of Cidofovir (an antiviral drug) and Rifabutin (an anti-tubercular drug) as anti-malarial agents.

### Run-time

**Figure 1:**
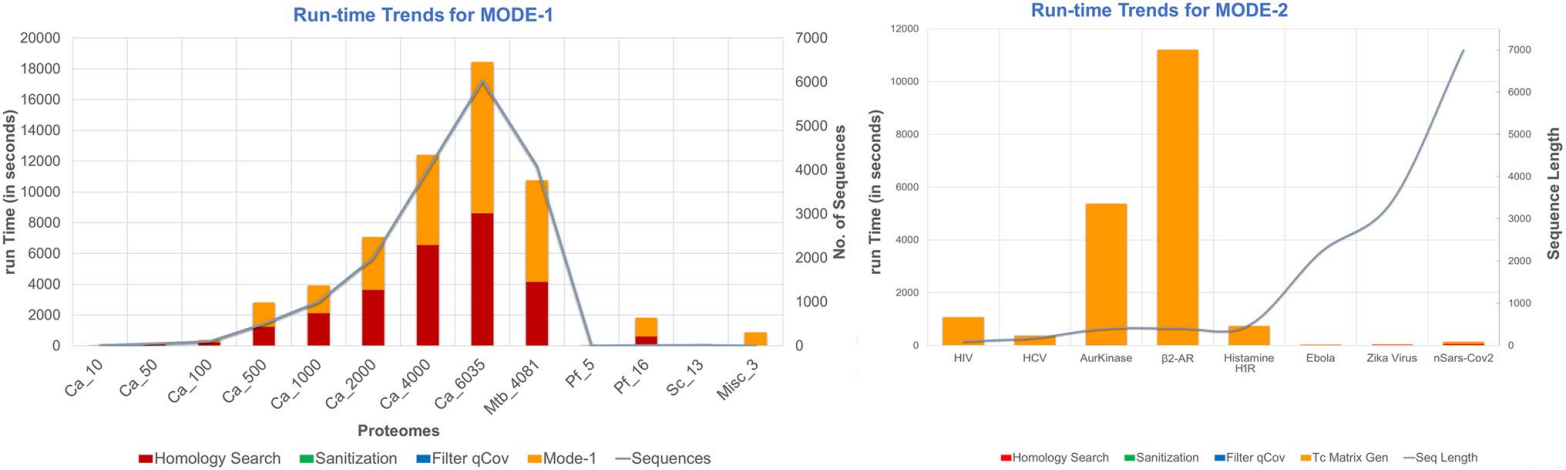
Performance statistics for MODE-1 and MODE-2 runs of NOD server with default settings. MODE-1 (left) is run against multiple sequence input data with different number of sequences (y_2_-axis) and run-time (y_1_-axis) is catalogued. With a gradual increase in No. of sequences (from 10 to 6035) using Candida albicans proteome, a non-linear increase in run-time is observed at Ca_1000 to Ca_2000. However, a comparison between the mode-1 runs of Ca_4000 and 4081 sequences of Mtb (Mtb_4081) shows that the run-time complexity is not essentially linear. It takes longer for Ca_4000 than Mtb_4081. Other protein sequence input includes: Plasmodium falciparum (Pf), nSARS-Cov2 (Sc), and miscellaneous set of 3 sequences comprising an Aurora Kinase and two GPCRs. For MODE-2 (right), it is evident that the computation time is independent of the sequence length as well. While Aurora-A Kinase (AurKinase), Beta-2 Adernergic Receptor (β_2_-AR) are relatively short sequences of 403 and 413 residues, their run-time ranges are very high in the order of 95 min to 197 min, respectively. Other sequences include: protease of HIV and HCV, RNA polymerase of Ebola, and Genome polyprotein of Zika virus.

In general, NOD is fast as the run-time varies in the order of few minutes. However, the run-time of any job is influenced by the number of QTC associations that are obtained. This suggests more the number of homologous target protein hits that a given query sequence fetches, the more are the number of DrugBank candidates associated with each target protein, and more will be the run-time. For example, a query sequence that is a homolog to popular drug targets, such as kinases and GPCRs, is expected to yield more associations and take a longer time to run than the sequences that are not well represented in the DrugBank target sequence space (Fig. 1).

### Browser compatibility

NOD has been tested and found to be compatible with popular internet browsers on all major desktop operating systems (Supplementary Table S3). We recommend that to enjoy all the features of NOD, users should access NOD through a desktop/laptop. The current version of NOD is not compatible with mobile browsers.

### New findings

Interestingly, the attempts to validate NOD results have generated lists of many compounds whose effectiveness in treating the respective disease has not yet been clinically investigated. Few examples include: Diacerein (current usage: osteoarthritis) against tuberculosis (predicted Mtb target: Lanosterol 14-alpha demethylase), Afatinib (anti-cancer agent) against candidiasis (predicted C_alb target: Cyclin-dependent kinase-1), and Tranilast (anti-allergic drug) against malaria (predicted P_fal target: Glutathione S-transferase). As discussed earlier, the control study too has led to the identification of potential compounds that could be considered for repurposing against diseases that are not known to be the primary indications of the suggested drugs (Table S2). Given the credibility of the predictions by NOD as evident from the validation studies, it is worth probing the novel associations (for other associations and details, kindly visit http://pauling.mbu.iisc.ac.in/NOD/NOD/webpages/archives.html).

A case study on SARS-CoV-2 proteins to demonstrate the use of NOD under both modes of operations could be found in the Supplementary Information (SI). The case study is illustrated with the step-by-step procedures that NOD follows to generate the results (Supplementary Fig. S1, S2; Table S4).

## Discussion

NOD web server is the first of its kind to aid drug repurposing with the following salient features: (i) fast, automated, and user-friendly; (ii) unlike most of the currently available resources, NOD exploits the information encoded in protein sequences rather than structures; (iii) performance is validated against available clinical and/or experimental data; (iv) uses a sensitive HMM-based sequence search strategy that facilitates better coverage of drug-target space.

Information on evolutionary relationship derived upon comparison of the input protein sequence/s with a set of pre-defined protein sequences (which are known to be targeted by DrugBank molecules) allows NOD to suggest a list of potential candidates which might interact with the queried protein/s. Homology detection between query and target database proteins is facilitated by a HMM-based iterative search^22^which offers an advantage over BLAST searches^23^ (as implemented in DrugBank) in identifying remote homologs. It is plausible that a pair of homologous proteins might have diverged extensively over the course of evolution, leaving no detectable similarity in overall sequence length except a local ligand-binding region. Under such circumstances, particularly the HMM-based sequence search methods help identify the remote homologs that share a very low sequence identity. These pairs of remote homologs generally remain undetected if simple BLAST searches are used. Hence, HMM-based search method implemented in NOD aims to minimize the chances of missing interesting candidate compounds. These compounds might interact with the queried protein which shares similarity with its remote target homologs in the ligand binding site. However, to appreciate the advantage of the HMM-based search implemented in NOD and detect interesting hits at a low sequence identity, one might need to lower the query coverage criterion below the default settings, i.e., 70%. Such a manipulation is advised for users who have ﻿expert understanding of their query protein/s. While lowering the cut-off below 70% may increase the chances of obtaining more hits, it will also increase the run-time for the job. Notably, under the MODE-1 operation, if a large number of query sequences are submitted by selecting a low query coverage cut-off, an unmanageably large number of hits might be obtained.

The three-dimensional (3-D) structure of a protein facilitates its biological function. The information on the structure of the protein is encoded in its sequence. Higher the sequence identity between two proteins, higher are the chances that the two proteins have similar structure and possibly a conserved ligand-binding site. If the ligand-binding site is outside the aligned region of two protein sequences, then the chances that these proteins will exhibit similar molecular recognition pattern are low. There are important measures in place to ensure NOD gives the most reliable query-target pairs and eventually suggests an associated compound that has high chances of interacting with the query protein. Only those query-target associations are reported where the aligned region contain at least 70% of the length of the query sequence detected with a E-value stringent than 10^–5^. The imposition of these selection criteria increases the confidence in the detected homologs. It ensures that the maximum length of the queried protein sequence is encompassed in the alignment, which is likely to span over the ligand-binding region. Users are encouraged to inspect the information on alignment 'start' and 'end' positions provided in result tables of NOD to confirm if the aligned regions of the protein sequences span over the known ligand binding region. Further, under physiological conditions, protein–ligand binding event is influenced by a host of other factors, such as shape, electrostatics, and flexibility of the two binding partners. These factors are beyond the scope of NOD to consider. The results from NOD shall be treated as starting points for any drug repurposing projects and thorough analysis is required on a case-by-case basis with integration of available experimental data. Previous reports from our group can be referred as examples of case-by-case sequence-guided drug repurposing investigations^18,19,21,39^. The hyperlinks to external databases such as UniProt^40^ and DrugBank in the result table of NOD provide important pointers for the users to mine relevant information on the query and target proteins and associated compounds. We encourage NOD users to exploit the information available in the mentioned external sources to conduct detailed study on the NOD suggested results.

NOD is programmed to handle any number of input sequences under MODE-1 of its operation. There is no upper limit on the number of sequences that can be submitted under MODE-1. MODE-2 deals with only a single input sequence and offers additional analysis to expand the chemical space, which is omputationally demanding. If a user is interested to subject multiple input sequences to MODE-2 analysis, it can be done by submitting each sequence of interest as a separate job. The major factor that determines the run time of NOD is the number of associations that a query protein sequence makes with the DrugBank molecules associated with each target homolog protein. The results compiled by NOD in the output tables are downloadable and can be easily parsed, allowing the user to perform personalized investigations. All these could be obtained in the order of a few minutes (for intensive jobs, it might take few hours) by just one-time submission of the query sequence(s).

NOD will be maintained and updated in the years to come following any major update of the DrugBank database. In the future versions of NOD, we plan to (i) introduce features that would help analyze the results on the web-interface and (ii) further improve the run-time of the jobs. Finally, it is challenging to arrange immediate resources for combating any unpredictable outbreaks of pandemics caused by novel pathogens. In this regard, we believe that NOD web server could be an essential component for preparing against any future unpredictable outbreak of novel pathogenic diseases when structures of drug targets in the pathogen may not be available.

## Methods

### Working Principle of NOD

NOD operates under two modes: MODE-1 and MODE-2. The NOD algorithm can be broadly divided into three modules/steps: (1) Sequence similarity search, (2) Mapping of compounds, and (3) Expansion of the chemical space. MODE-1 and MODE-2 partly shares the same workflow (step -1 and step-2) as detailed later. The step-3, i.e., expansion of chemical space, is exclusive to MODE-2 of NOD. The two modes of NOD are intended to serve different purposes. The MODE-1 of the pipeline accepts multiple input sequences and would be helpful in facilitating generic searches. For example, when the user is interested in finding potential repurpose-able candidates against all proteins in a given organism can provide the entire proteome of the organism as query. The MODE-2 of the pipeline accepts a single protein sequence and is intended to serve specific investigational needs. Besides suggesting potential repurpose-able candidates against the input protein, the MODE-2 of NOD allows expansion of the chemical space of the suggested candidates through similarity searches within the library of FDA-approved drugs. Combination of MODE-1 and MODE-2 operations would be helpful when users start with a general investigation on multiple protein sequences (MODE-1) and then focusses on specific query proteins of interest (MODE-2) for further analyses based on the outcomes from MODE-1. The two modes of operations are independent of each other. Therefore, depending upon the questions to address, users can even choose to submit jobs in either of the two modes alone by using the designated forms available on the NOD web-interface.

**Figure 2:**
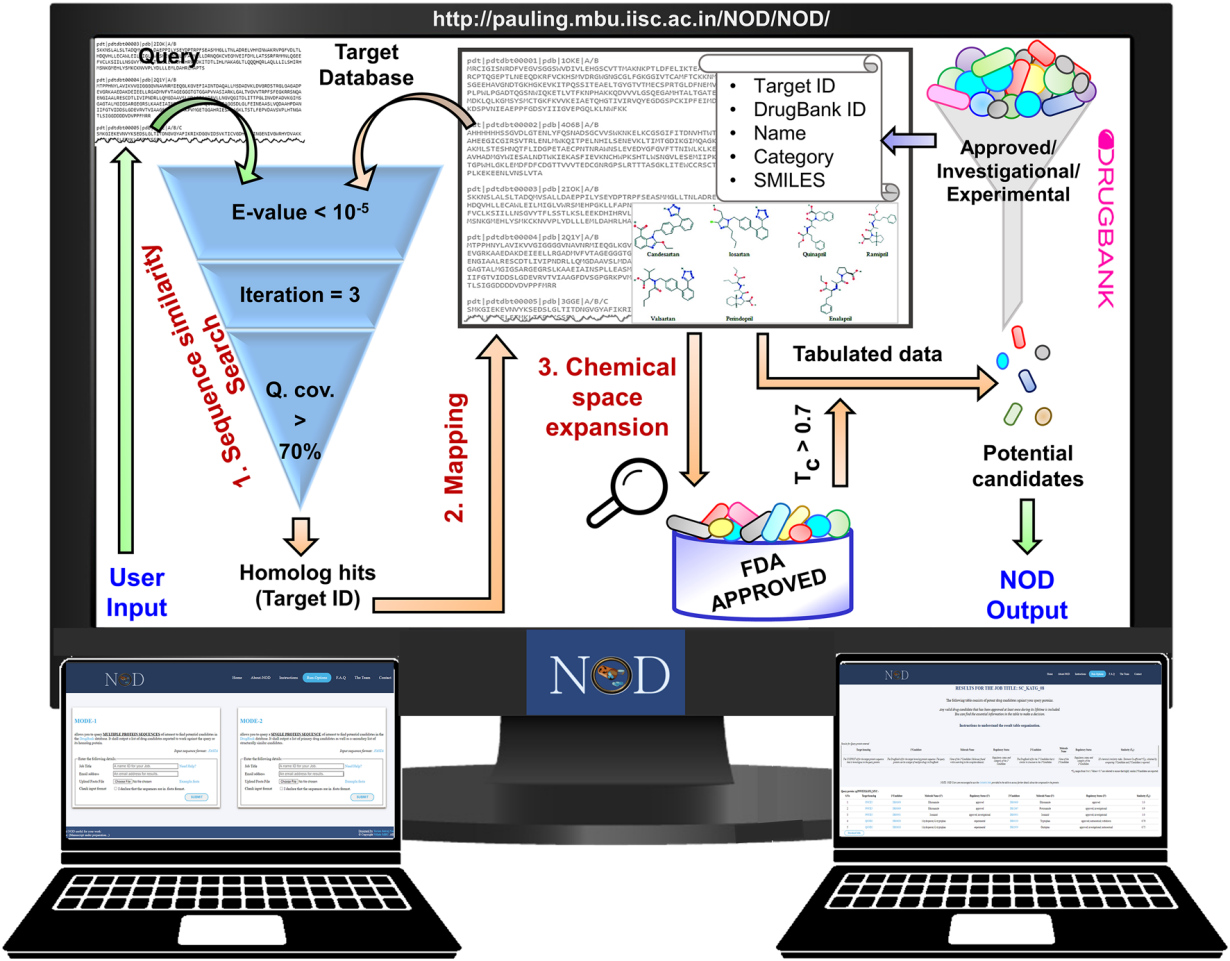
Workflow involved in NOD pipeline. Steps—1 and 2 are common to both MODE-1 and 2. Step 3 is executed only in MODE-2. The user submits the input sequence/s (query) using the forms available in ‘Run-Options’ page of NOD server. The homologs of the query protein/s are then searched and subsequently the relevant information of associated compounds is fetched to suggest a list of potential candidates which could be considered for further probing in the drug repurposing pipeline. MODE-2 offers an additional analysis by allowing an expansion of the primary chemical space through similarity search quantified using Tanimoto coefficient (T_c_). Q. cov. indicates Query coverage.

The steps involved in NOD's operations are elaborated below (Fig. 2).

1. **Sequence similarity search**: NOD employs an iterative (3 tiered) HMM-based sequence search implemented in the Jackhmmer module from HMMER3.3^41^ suite to identify closely and distantly related proteins in a pre-defined target database of protein sequences. The hits which satisfy the following conditions: (a) E-value < 10^–5^ and (b) an alignment length covering > 70% (default) of the region in the queried sequence, are considered reliable hits. The current version of NOD does not allow to lower the query coverage below 40% under MODE-1. Lowering the query coverage below 40% or completely ignoring the criterion is possible under MODE-2 based on the user's discretion.
2. **Mapping of compounds**: The target database of protein sequences against which the input sequence/s is queried has been derived from DrugBank version 5.1.7^42^. One or more known compounds target these proteins. These are either approved therapeutic agents or are at various stages of clinical investigations/ pre-clinical studies. Hereafter, this DrugBank database of compounds is referred to as the 'all' dataset. Upon establishing a reliable evolutionary relationship between a query and target database protein, the information on DrugBank compounds linked with the target homolog protein is fetched. These compounds are then linked with the query protein/s. They may interact with the query protein by virtue of their similarity to the target protein and can be potential candidates for repurposing against the queried protein.

While the above two steps are common to both modes, the step described below is exclusive to MODE-2 of NOD.

3. **Expansion of the chemical space**: The potential compounds (primary candidates) associated with the query protein in the previous step are used as a query to search for chemically similar compounds in the subset of approved drugs. Tanimoto coefficient (T_c_), a similarity index, is calculated between the DrugBank compounds associated with the target homolog proteins and the drugs in the approved library^43^. It is known that compounds with similar chemical functionalities may elicit similar biological activity^44^. Hence, compounds (secondary candidates) with T_c_ > 0.7, i.e., > 70% similarity in the chemical 2-D structures, are included in the final list to ensure that only the compounds which are highly similar to the primary candidates are reported. It is worth mentioning that failing to obtain a secondary candidate indicates that no approved compound is more than 70% similar to the primary candidate suggested by NOD. In such scenarios, the unique chemical structure of the primary candidate can be exploited further in the drug development pipeline.
  The additional analysis of chemical space expansion is not offered by MODE-1, which is intended to handle a large number of sequences. This ensures a manageable set of useful results from MODE-1 jobs within a reasonable time for immediate investigations by the users.

### NOD web server implementation

The NOD web server comprises three layers: (a) Web interface, (b) Algorithm, and (c) Communication interface. NOD back-end algorithm is scripted in Python3.8, while the front-end web application is designed in HTML5 (supported by javascript). A detailed description of the three layers is presented below.

- **Web Interface:** The layer comprises HTML5 webpages that are styled using CSS (stylesheets). The pages are made responsive using javascript. A user would interact exclusively with this layer to get acquainted with the NOD pipeline and submit job/s to initiate the pipeline.
  i. *Home Page*: The landing page for NOD is the home page, which provides a brief overview of drug repurposing. This page also provides access to other pages of the application via the top navigation panel.
  ii. *About NOD:* It has dedicated information about the pipeline, its different modules, and the technology used to develop NOD. Further, a responsive subsection: 'The Pipeline', which comprises all the technical details about NOD algorithm, is provided in line with our open access principle.
  iii. *Instructions:* A clear set of instructions are provided for the user to operate NOD. The first two responsive subsections detail the two modes of operations. These sections provide useful example scenarios that would guide a user in deciding the mode of operations and the subsequent result interpretations. The third responsive section educates the user about the scenarios where the user's discretion is of prime importance while analyzing the NOD output.
  iv. *Run*-*Options:* Once the user is acquainted with the basic philosophy of drug repurposing and the NOD pipeline through the first three pages, the fourth page in the NOD web-interface allows the users to submit jobs using the respective forms. A valid email address needs to be provided in the form to which the links to the result file obtained from the NOD run will be sent.
  v. *Arch*-*NOD:* This page archives the results of all model cases that we, the developers, have run on the NOD server as a part of the validation efforts and control studies to evaluate the performance of NOD.
  vi. *F.A.Q:* This page contains a set of frequently asked questions that are descriptively answered to help users troubleshoot any problem while running the pipeline.
  vii. *Contact:* The form can be used to raise concerns about any job or provide constructive feedback for making NOD perform better. In either case, a member of the NOD team would respond within one working day.
- **Algorithm:** It forms the core of the pipeline and comprises a program written in Python_v3.8. It identifies related proteins and extracts relevant information from the NOD-integrated database derived from Drugbank^42^ and handles all the inputs and outputs when the pipeline runs. The algorithm is programmed to perform four major tasks:
-
  i. *Authentication and homology search*: Examine input sequence/s, convert them into NOD-friendly formats, and perform the homology search using JackHmmer v3.3^41^.
  ii. *Sanitization and filtering*: The JackHmmer search results are formatted in an easily parse-able structure followed by filtering reliable hits based on the supplied alignment coverage cut-off (default is 70% of query sequence length; otherwise, a user-defined coverage cut-off is used).
  iii. *Mapping and similarity calculation*: Extract the list of Drugbank compounds associated with the homologous target protein/s and other relevant information. If the pipeline is operated under Mode-2, the next step (iii-a) is performed; else, the control passes on to the compilation and reporting module. iii(a)*.* In Mode-2, an additional step to compare the chemical similarity (measured in terms of T_c_) between pair of compounds is performed using the RDKit library (http://www.rdkit.org). Chemical fingerprints of the primary candidates from the 'all' dataset and each compound in the 'approved' subset are generated from the respective SMILES code^45^ to calculate T_c_. The compounds with T_c_ > 0.7 are considered for final reporting. These are the secondary candidates.
  iv. *Compilation and reporting*: Report the compiled results in the form of python Pandas^46^ dataframes for quick processing. The entries in the result table are sorted based on descending query coverage values.
- **Communication interface:** Written using Python CGI and MIME mailing libraries, the code's sole responsibility is to communicate between the front-end and back-end layers.

The NOD source-code is openly available at https://github.com/WebServer-NOD-Discussions/NOD-git.git.

## Supplementary Information

Supplementary Information 1.

Supplementary Information 2.

Supplementary Information 3.
