# Supplementary material for: NOD: a web server to predict New use of Old Drugs to facilitate drug repurposing
Source: Sci Rep. 2021 Jun 29;11:13540. doi: 10.1038/s41598-021-92903-8 (PMC8241987; doi:10.1038/s41598-021-92903-8)
Supplement: Supplementary file 1 — Supplementary Information 1. [file 41598_2021_92903_MOESM1_ESM.docx]

**Supplementary Information**

**NOD: A web server to predict New use of Old Drugs to facilitate drug repurposing**

Tarun J. Narwani^a^, Narayanaswamy Srinivasan^a*^, Sohini Chakraborti^a^

^a^Molecular Biophysics Unit, Indian Institute of Science, Bengaluru 560012, Karnataka, India.

*To whom correspondence should be addressed: [ns@iisc.ac.in](mailto:ns@iisc.ac.in)

**Results**

**Table S3: Details of browser compatibility test**

| **S. No.** | **Desktop operating system** | **Internet browser** | **Are all the features of NOD accessible?** |
| --- | --- | --- | --- |
| 1 | Windows 10 | Google chrome ver. 86.0.x.x | Yes |
| 2 | Windows 10 | Microsoft edge ver. 86.0.x.x | Yes |
| 3 | Windows 10 | Firefox ver. 83.0.x | Yes |
| 4 | Windows 10 | Opera ver. 66 | Yes |
| 5 | Macintosh | Safari ver. 13.1 | Yes |
| 6 | Macintosh | Firefox ver. 82.0.x | Yes |
| 7 | Ubuntu 16.04 | Firefox ver. 82.0.x | Yes |
| 8 | Ubuntu 16.04 | Google chrome ver. 74.0.x | Yes |

**Case study**

A case study with SARS-CoV-2 proteins:

**MODE-1:** The protein sequences of SARS-CoV-2 (the causative agent for Covid-19) corresponding to the reviewed entries in UniProt^1^ were retrieved and submitted to NOD. 16 SARS-CoV-2 protein sequences had ‘reviewed’ status in the UniProt at the time of conducting this study. These 16 sequences include the proteins like replicase polyprotein 1ab, spike glycoprotein, M protein, nucleoprotein etc. (for details, see Table S4). Such an all-inclusive approach where multiple (or even all) proteins of a pathogen are queried, minimizes the chances of missing any interesting candidate compound that might have the potential to be repurposed against any protein target implicated in the disease of interest. NOD could find reliable homologs for 2 out of the 16 queried SARS-CoV-2 proteins from the DrugBank target sequence database. Subsequently, 20 unique query-target-compound (QTC) associations were generated by NOD, and the entire job was completed in just 285.89 seconds. Fig. S1 shows a step-by-step flow of events on NOD web-interface upon submission of a job under MODE-1 operation. As could be seen in the fifth step of Fig. S1, NOD identified known antiviral agents like remdesivir and GS-441524, which are reported to be effective against coronavirus infections^2,3^. These compounds are potential candidates that may be probed for repurposing against replicase polyprotein 1a (UniProt accession code: P0DTC1) of SARS-CoV-2 based on its detected homology with replicase polyprotein 1ab (UniProt accession code: P0C6X7) of SARS-CoV. The alignment between the two protein sequences (query and target) spans over almost the entire length (99.9%) of the query protein, ensuring the similarity of the ligand-binding sites between the two proteins. Excitingly, reports from various research groups, including ours, have discussed the potential of remdesivir in treating SARS-CoV-2 infection^2,4^, and it is currently being probed under various clinical trials to explore it's usage in anti-Covid19 therapy and has also been approved for emergency usage in many countries. The details on clinical trials of remdesivir could be found at [https://www.clinicaltrials.gov/ct2/results?recrs=&cond=&term=remdesivir&cntry=&state=
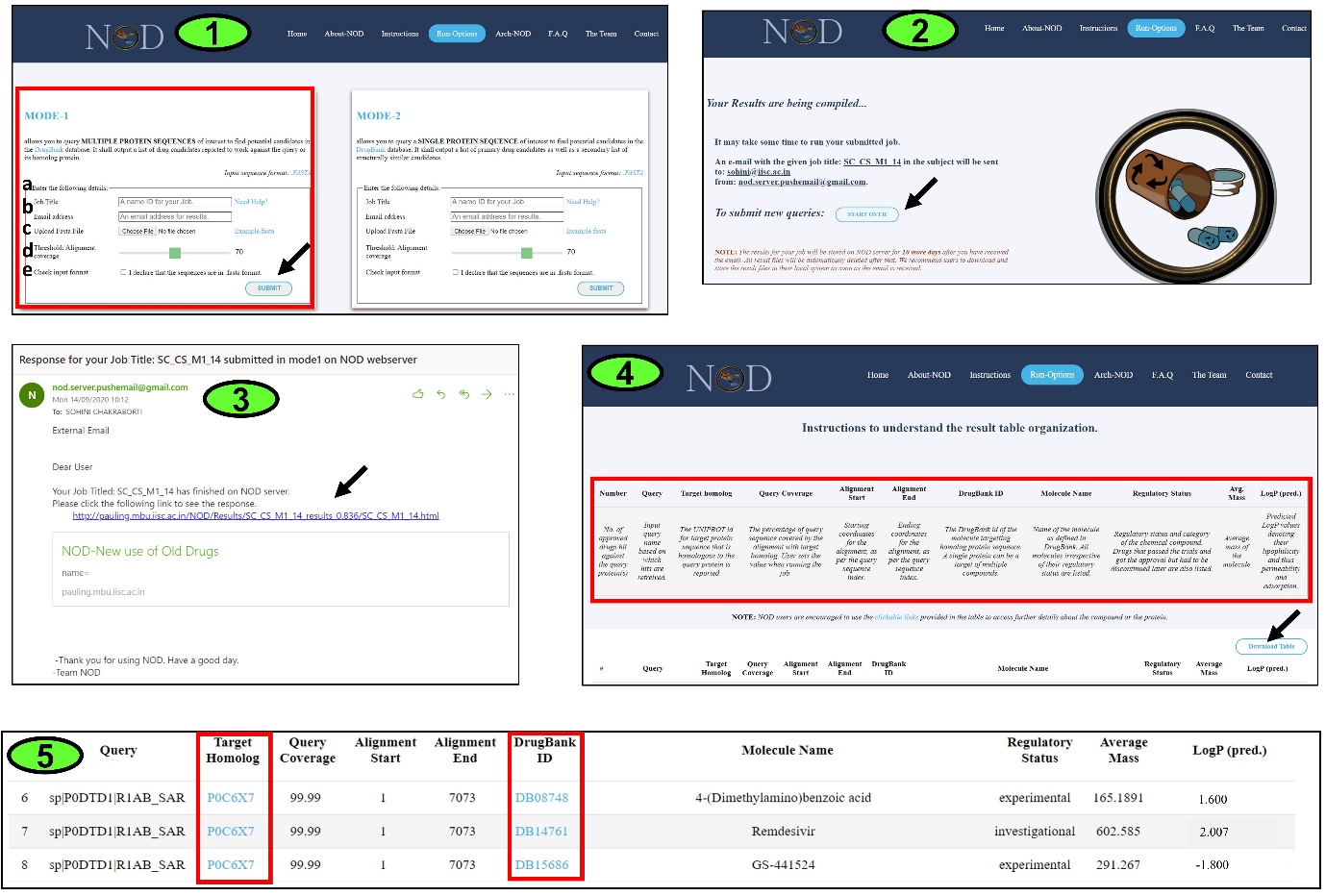
&city=&dist=](https://www.clinicaltrials.gov/ct2/results?recrs=&cond=&term=remdesivir&cntry=&state=#&city=&dist=).

**Fig. S1: A step-by-step flow of events under MODE-1 operation of NOD depicted with screen shots from the example case study on SARS-CoV-2 proteins.** (1) Jobs can be submitted to NOD by hitting the ‘SUBMIT’ button (indicated by a black arrow) after filling up the details in the desired mode available under ‘Run-Options’ page. The details required are: (a) Job Title, (b) E-mail address (where the link to the downloadable result table obtained as output from NOD will be sent), (c) A file containing the protein sequences in ‘.FASTA’ format needs to be uploaded, (d) Adjusting the Query coverage cut-off (if required), and (e) Declaration that the submitted sequences conform to ‘.FASTA’ format (which is the NOD-friendly format). (2) Upon successful job submission, a window opens which informs the user that the responses from NOD will be notified to the provided e-mail address. New jobs can be submitted by clicking on the ‘START OVER’ button (indicated in black arrow) which would take the user to the ‘Run-Options’ page. (3) Once the job is completed, an automated e-mail is sent by the NOD server to the user supplied e-mail ID with a link (indicated with black arrow) to view the responses from NOD. (4) On clicking the link, a HTML file with tabulated data is displayed on the NOD interface. Description on the type of data compiled in each column is provided at the top of the table (highlighted with a red box). The table can be downloaded as a tab-separated file by clicking on the ‘Download Table’ button (indicated with a black arrow). The download-able table contains additional information derived from the DrugBank database (for example, name of the target protein, SMILES code of the shortlisted compounds, etc.). The result tables are automatically deleted from the NOD server after 10 days. (5) A snapshot of the results from the output table has been shown here. The UniProt accession code of the target homologue protein and the DrugBank ID of the candidate molecule are provided as clickable links (highlighted with red box) which can direct the user to the respective databases for more details.

**Table S4: List of SARS-CoV-2 protein sequences submitted to NOD under MODE-1 test operation**

| **Sl. No.** | **UniProt Code** | **Name of the protein** |
| --- | --- | --- |
| 1 | P0DTD1 | **Replicase polyprotein 1ab** |
| 2 | P0DTC2 | Spike glycoprotein |
| 3 | P0DTC1 | **Replicase polyprotein 1a** |
| 4 | P0DTC7 | **ORF7a protein** |
| 5 | P0DTC3 | **ORF3a protein** |
| 6 | P0DTC5 | **Membrane protein** |
| 7 | P0DTC9 | **Nucleoprotein** |
| 8 | P0DTD2 | **ORF9b protein** |
| 9 | P0DTC6 | **ORF6 protein** |
| 10 | P0DTC4 | **Envelope small membrane protein** |
| 11 | P0DTC8 | **ORF8 protein** |
| 12 | P0DTD8 | ORF7b protein |
| 13 | P0DTD3 | ORF9c protein |
| 14 | P0DTF1 | ORF3b protein |
| 15 | P0DTG0 | ORF3d protein |
| 16 | P0DTG1 | ORF3c protein |

**MODE-2:** The amino acid sequence of replicase polyprotein 1ab in SARS-CoV-2 (P0DTD1, https://www.uniprot.org/uniprot/P0DTD1) was used as input in MODE-2. The sequence of this protein encodes multiple proteins of the virus, including the proteases which are responsible for cleavage of the polyprotein to functional forms, thereby aiding in the process of viral transcription and replication. One of these proteases is the 3C-like protease, or the main protease, which plays a crucial role in the viral life-cycle and is an important drug target^5^. The homolog of SARS-CoV-2 replicase polyprotein 1ab as detected by NOD in the DrugBank database, is SARS-CoV replicase polyprotein 1ab with 99.9% alignment coverage for the query sequence. Recently we have reported potential candidates that can be probed for repurposing against the SARS-CoV-2 main protease primarily using computational structure-guided approaches^4^. Interestingly, some of our earlier reported candidates could also be found in the results generated by NOD that employs a sequence-guided approach followed by 2-D chemical similarity search under its MODE-2 operation. The notable hits are the vinca alkaloids (vinorelbine, vincristine, vindesine, etc.) and known antiviral agent, baloxavir marboxil (Fig.S2).


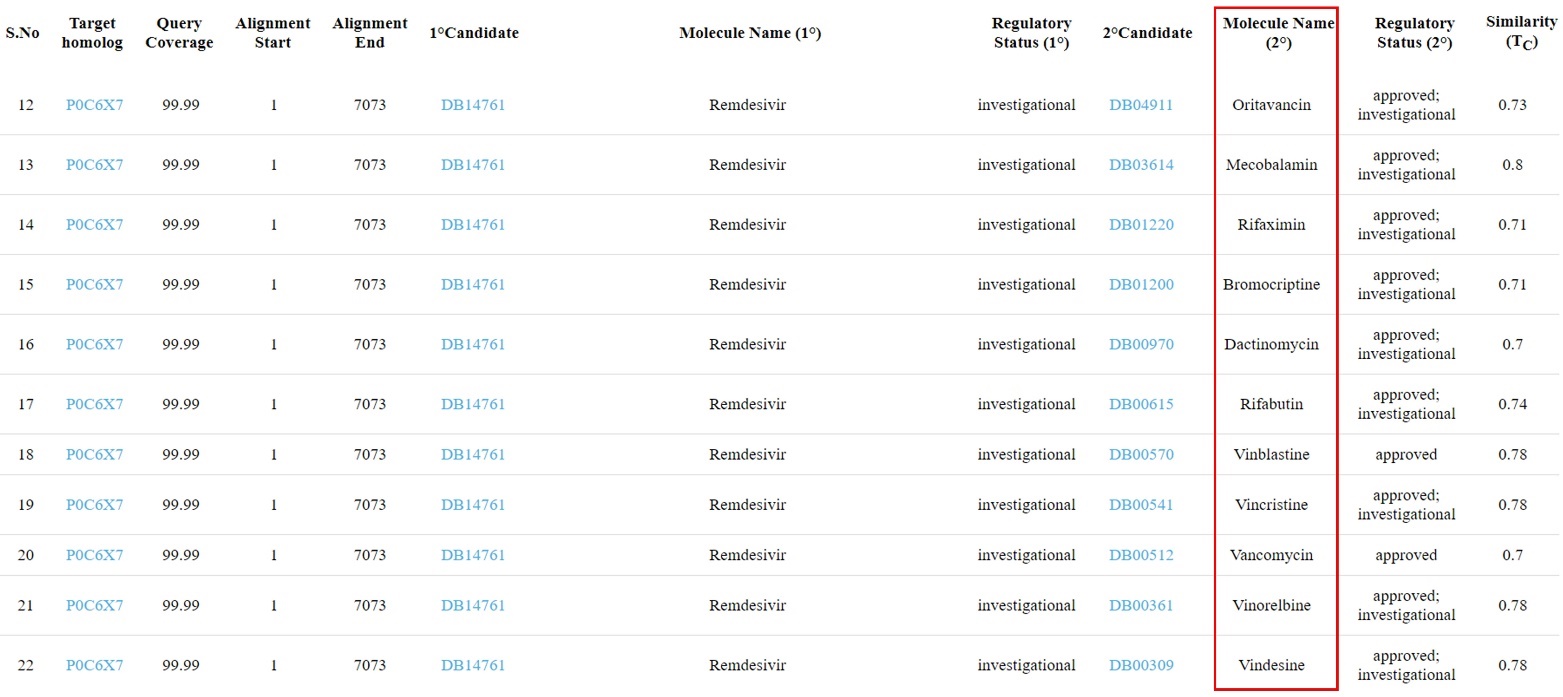


**Fig. S2: Snapshot of results from SARS-CoV-2 MODE-2 job.** The column highlighted within the red rectangle contains the secondary candidates obtained from similarity search. Several vinca alkaloids (like vinorelbine, vincristine, vindesine etc.) and known antiviral agent are reported by NOD as discussed in the text. Full result for this job could be viewed at http://pauling.mbu.iisc.ac.in/NOD/NOD/webpages/archives.html.

P.S. : Apart from the references cited in the text of the supplementary information, the list below also includes all the references cited in the supplementary tables S1 and S2 (.xlsx files).

**References**

1. Consortium, T. U. UniProt: a worldwide hub of protein knowledge. *Nucleic Acids Res.* **47**, D506–D515 (2018).

2. Wang, Y. *et al.* Remdesivir in adults with severe COVID-19: a randomised, double-blind, placebo-controlled, multicentre trial. *Lancet* **395**, 1569–1578 (2020).

3. Amirian, E. S. & Levy, J. K. Current knowledge about the antivirals remdesivir (GS-5734) and GS-441524 as therapeutic options for coronaviruses. *One Heal.* **9**, 100128 (2020).

4. Chakraborti, S., Bheemireddy, S. & Srinivasan, N. Repurposing drugs against the main protease of SARS-CoV-2: mechanism-based insights supported by available laboratory and clinical data. *Mol. Omi.* (2020) doi:10.1039/D0MO00057D.

5. Amin, S. A., Banerjee, S., Ghosh, K., Gayen, S. & Jha, T. Protease targeted COVID-19 drug discovery and its challenges: Insight into viral main protease (Mpro) and papain-like protease (PLpro) inhibitors. *Bioorg. Med. Chem.* 115860 (2020) doi:10.1016/j.bmc.2020.115860.

6. Maddix, D. S., Tallian, K. B. & Mead, P. S. Rifabutin: a review with emphasis on its role in the prevention of disseminated Mycobacterium avium complex infection. *Ann. Pharmacother.* **28**, 1250–1254 (1994).

7. Floss, H. G. & Yu, T.-W. Rifamycin-mode of action, resistance, and biosynthesis. *Chem. Rev.* **105**, 621–632 (2005).

8. Ho, Y. I., Chan, C. Y. & Cheng, A. F. In-vitro activities of aminoglycoside-aminocyclitols against mycobacteria. *J. Antimicrob. Chemother.* **40**, 27–32 (1997).

9. DeBarber, A. E., Mdluli, K., Bosman, M., Bekker, L. G. & Barry, C. E. 3rd. Ethionamide activation and sensitivity in multidrug-resistant Mycobacterium tuberculosis. *Proc. Natl. Acad. Sci. U. S. A.* **97**, 9677–9682 (2000).

10. Vilchèze, C. & Jacobs, W. R. J. The mechanism of isoniazid killing: clarity through the scope of genetics. *Annu. Rev. Microbiol.* **61**, 35–50 (2007).

11. Xu, J. *et al.* Contribution of Pretomanid to Novel Regimens Containing Bedaquiline with either Linezolid or Moxifloxacin and Pyrazinamide in Murine Models of Tuberculosis. *Antimicrob. Agents Chemother.* **63**, (2019).

12. Goude, R., Amin, A. G., Chatterjee, D. & Parish, T. The arabinosyltransferase EmbC is inhibited by ethambutol in Mycobacterium tuberculosis. *Antimicrob. Agents Chemother.* **53**, 4138–4146 (2009).

13. Campbell, E. A. *et al.* Structural mechanism for rifampicin inhibition of bacterial rna polymerase. *Cell* **104**, 901–912 (2001).

14. Palomino, J. C. & Martin, A. The potential role of trimethoprim-sulfamethoxazole in the treatment of drug-resistant tuberculosis. *Future Microbiol.* **11**, 539–547 (2016).

15. Koul, A. *et al.* Diarylquinolines target subunit c of mycobacterial ATP synthase. *Nat. Chem. Biol.* **3**, 323–324 (2007).

16. Sterling, T. R. *et al.* Three months of rifapentine and isoniazid for latent tuberculosis infection. *N. Engl. J. Med.* **365**, 2155–2166 (2011).

17. Vandekerckhove, S. & D’hooghe, M. Quinoline-based antimalarial hybrid compounds. *Bioorg. Med. Chem.* **23**, 5098–5119 (2015).

18. Olliaro, P. L., Haynes, R. K., Meunier, B. & Yuthavong, Y. Possible modes of action of the artemisinin-type compounds. *Trends Parasitol.* **17**, 122–126 (2001).

19. Fuchs, A. *et al.* Falciparum malaria-induced secondary hemophagocytic lymphohistiocytosis successfully treated with ruxolitinib. *International journal of infectious diseases : IJID : official publication of the International Society for Infectious Diseases* (2020) doi:10.1016/j.ijid.2020.07.062.

20. Kobylinski, K. C. *et al.* Rationale for the coadministration of albendazole and ivermectin to humans for malaria parasite transmission control. *Am. J. Trop. Med. Hyg.* **91**, 655–662 (2014).

21. Wiesner, J., Borrmann, S. & Jomaa, H. Fosmidomycin for the treatment of malaria. *Parasitol. Res.* **90 Suppl 2**, S71-6 (2003).

22. Kaneko, A. *et al.* Intrinsic efficacy of proguanil against falciparum and vivax malaria independent of the metabolite cycloguanil. *J. Infect. Dis.* **179**, 974–979 (1999).

23. Stauffer, W. & Fischer, P. R. Diagnosis and treatment of malaria in children. *Clin. Infect. Dis. an Off. Publ. Infect. Dis. Soc. Am.* **37**, 1340–1348 (2003).

24. Barnes, K. I. *et al.* Sulfadoxine-pyrimethamine pharmacokinetics in malaria: Pediatric dosing implications. *Clin. Pharmacol. Ther.* **80**, 582–596 (2006).

25. Crowley, P. D. & Gallagher, H. C. Clotrimazole as a pharmaceutical: past, present and future. *J. Appl. Microbiol.* **117**, 611–617 (2014).

26. Vazquez, J. A. Anidulafungin: a new echinocandin with a novel profile. *Clin. Ther.* **27**, 657–673 (2005).

27. Groll, A. H. & Walsh, T. J. Posaconazole: clinical pharmacology and potential for management of fungal infections. *Expert Rev. Anti. Infect. Ther.* **3**, 467–487 (2005).

28. Falci, D. R. & Pasqualotto, A. C. Profile of isavuconazole and its potential in the treatment of severe invasive fungal infections. *Infect. Drug Resist.* **6**, 163–174 (2013).

29. McCormack, P. L. & Perry, C. M. Caspofungin: a review of its use in the treatment of fungal infections. *Drugs* **65**, 2049–2068 (2005).

30. Groll, A. H., Stergiopoulou, T., Roilides, E. & Walsh, T. J. Micafungin: pharmacology, experimental therapeutics and clinical applications. *Expert Opin. Investig. Drugs* **14**, 489–509 (2005).

31. Patel, T. & Dhillon, S. Efinaconazole: first global approval. *Drugs* **73**, 1977–1983 (2013).

32. Vanden Bossche, H. & Marichal, P. Mode of action of anti-Candida drugs: focus on terconazole and other ergosterol biosynthesis inhibitors. *Am. J. Obstet. Gynecol.* **165**, 1193–1199 (1991).

33. Toledo-Bahena, M. E. *et al.* The efficacy and safety of tavaborole, a novel, boron-based pharmaceutical agent: phase 2 studies conducted for the topical treatment of toenail onychomycosis. *J. Drugs Dermatol.* **13**, 1124–1132 (2014).

34. Iwatani, W., Arika, T. & Yamaguchi, H. Two mechanisms of butenafine action in Candida albicans. *Antimicrob. Agents Chemother.* **37**, 785–788 (1993).

35. Thomason, J. L. Clinical evaluation of terconazole. United states experience. *J. Reprod. Med.* **34**, 597–601 (1989).

36. Khalandi, H. *et al.* Antifungal Activity of Capric Acid, Nystatin, and Fluconazole and Their In Vitro Interactions Against Candida Isolates from Neonatal Oral Thrush. *Assay Drug Dev. Technol.* **18**, 195–201 (2020).

37. Zhu, J., Luther, P. W., Leng, Q. & Mixson, A. J. Synthetic histidine-rich peptides inhibit Candida species and other fungi in vitro: role of endocytosis and treatment implications. *Antimicrob. Agents Chemother.* **50**, 2797–2805 (2006).

38. Flucytosine. in (ed. Aronson, J. K. B. T.-M. S. E. of D. (Sixteenth E.) 355–358 (Elsevier, 2016). doi:https://doi.org/10.1016/B978-0-444-53717-1.00753-8.

39. Smith, W. L. & Edlind, T. D. Histone deacetylase inhibitors enhance Candida albicans sensitivity to azoles and related antifungals: correlation with reduction in CDR and ERG upregulation. *Antimicrob. Agents Chemother.* **46**, 3532–3539 (2002).

40. Hoberg, K. A., Cihlar, R. L. & Calderone, R. A. Inhibitory effect of cerulenin and sodium butyrate on germination of Candida albicans. *Antimicrob. Agents Chemother.* **24**, 401–408 (1983).

41. Aeed, P. A., Young, C. L., Nagiec, M. M. & Elhammer, A. P. Inhibition of inositol phosphorylceramide synthase by the cyclic peptide aureobasidin A. *Antimicrob. Agents Chemother.* **53**, 496–504 (2009).

42. Kaneko, Y., Ohno, H., Imamura, Y., Kohno, S. & Miyazaki, Y. The effects of an hsp90 inhibitor on the paradoxical effect. *Jpn. J. Infect. Dis.* **62**, 392–393 (2009).

43. Hernández Molina, J. M., Llosá, J. & Ventosa, A. In vitro activity of nitroxoline against clinical isolates of Candida species. *Mycoses* **34**, 323–325 (1991).

44. Ryder, N. S., Frank, I. & Dupont, M. C. Ergosterol biosynthesis inhibition by the thiocarbamate antifungal agents tolnaftate and tolciclate. *Antimicrob. Agents Chemother.* **29**, 858–860 (1986).

45. Thierbach, G. & Reichenbach, H. Myxothiazol, a new antibiotic interfering with respiration. *Antimicrob. Agents Chemother.* **19**, 504–507 (1981).

46. Bonifaz, A. *et al.* The efficacy and safety of sertaconazole cream (2 %) in diaper dermatitis candidiasis. *Mycopathologia* **175**, 249–254 (2013).

47. Vila, T., Ishida, K., Seabra, S. H. & Rozental, S. Miltefosine inhibits Candida albicans and non-albicans Candida spp. biofilms and impairs the dispersion of infectious cells. *Int. J. Antimicrob. Agents* **48**, 512–520 (2016).

48. Dischler, N. M. *et al.* Wortmannin and Wortmannine Analogues from an Undescribed Niesslia sp. *J. Nat. Prod.* **82**, 532–538 (2019).

49. Beggs, W. H. Fungicidal activity of tioconazole in relation to growth phase of Candida albicans and Candida parapsilosis. *Antimicrob. Agents Chemother.* **26**, 699–701 (1984).

50. Taghipour, S. *et al.* Luliconazole, a new antifungal against Candida species isolated from different sources. *J. Mycol. Med.* **28**, 374–378 (2018).

51. Ryder, N. S. Terbinafine: mode of action and properties of the squalene epoxidase inhibition. *Br. J. Dermatol.* **126 Suppl**, 2–7 (1992).

52. Chen, Z. *et al.* Synergistic Activity of Econazole-Nitrate and Chelerythrine against Clinical Isolates of Candida albicans. *Iran. J. Pharm. Res. IJPR* **13**, 567–573 (2014).

53. Yamaguchi, H., Hiratani, T. & Plempel, M. In vitro studies of a new imidazole antimycotic, bifonazole, in comparison with clotrimazole and miconazole. *Arzneimittelforschung.* **33**, 546–551 (1983).

54. Niewerth, M. *et al.* Ciclopirox olamine treatment affects the expression pattern of Candida albicans genes encoding virulence factors, iron metabolism proteins, and drug resistance factors. *Antimicrob. Agents Chemother.* **47**, 1805–1817 (2003).

55. Lima, T. C., Ferreira, A. R., Silva, D. F., Lima, E. O. & de Sousa, D. P. Antifungal activity of cinnamic acid and benzoic acid esters against Candida albicans strains. *Nat. Prod. Res.* **32**, 572–575 (2018).

56. Polak, A. Oxiconazole, a new imidazole derivative. Evaluation of antifungal activity in vitro and in vivo. *Arzneimittelforschung.* **32**, 17–24 (1982).

57. Fontenelle, R. O. S. *et al.* Alkylphenol Activity against Candida spp. and Microsporum canis: A Focus on the Antifungal Activity of Thymol, Eugenol and O-Methyl Derivatives. *Molecules* **16**, 6422–6431 (2011).

58. Gebremedhin, S., Dorocka-Bobkowska, B., Prylinski, M., Konopka, K. & Duzgunes, N. Miconazole activity against Candida biofilms developed on acrylic discs. *J. Physiol. Pharmacol. an Off. J. Polish Physiol. Soc.* **65**, 593–600 (2014).

59. Farber, B. F. & Wolff, A. G. Salicylic acid prevents the adherence of bacteria and yeast to silastic catheters. *J. Biomed. Mater. Res.* **27**, 599–602 (1993).

60. Lei, J., Xu, J. & Wang, T. In vitro susceptibility of Candida spp. to fluconazole, itraconazole and voriconazole and the correlation between triazoles susceptibility: Results from a five-year study. *J. Mycol. Med.* **28**, 310–313 (2018).

61. Ceberio, I. *et al.* Safety of voriconazole and sirolimus coadministration after allogeneic hematopoietic SCT. *Bone Marrow Transplant.* **50**, 438–443 (2015).

62. Daneshmend, T. K. & Warnock, D. W. Clinical pharmacokinetics of ketoconazole. *Clin. Pharmacokinet.* **14**, 13–34 (1988).

63. Lu, I.-L. *et al.* Structure-Based Drug Design and Structural Biology Study of Novel Nonpeptide Inhibitors of Severe Acute Respiratory Syndrome Coronavirus Main Protease. *J. Med. Chem.* **49**, 5154–5161 (2006).

64. Verschueren, K. H. G. *et al.* A structural view of the inactivation of the SARS coronavirus main proteinase by benzotriazole esters. *Chem. Biol.* **15**, 597–606 (2008).

65. Bacha, U. *et al.* Development of Broad-Spectrum Halomethyl Ketone Inhibitors Against Coronavirus Main Protease 3CLpro. *Chem. Biol. Drug Des.* **72**, 34–49 (2008).

66. Goetz, D. H. *et al.* Substrate Specificity Profiling and Identification of a New Class of Inhibitor for the Major Protease of the SARS Coronavirus,. *Biochemistry* **46**, 8744–8752 (2007).

67. Hoetelmans, R. M. *et al.* Clinical pharmacology of HIV protease inhibitors: focus on saquinavir, indinavir, and ritonavir. *Pharm. World Sci.* **19**, 159–175 (1997).

68. Hull, M. W. & Montaner, J. S. G. Ritonavir-boosted protease inhibitors in HIV therapy. *Ann. Med.* **43**, 375–388 (2011).

69. Sadler, B. M., Hanson, C. D., Chittick, G. E., Symonds, W. T. & Roskell, N. S. Safety and pharmacokinetics of amprenavir (141W94), a human immunodeficiency virus (HIV) type 1 protease inhibitor, following oral administration of single doses to HIV-infected adults. *Antimicrob. Agents Chemother.* **43**, 1686–1692 (1999).

70. Luna, B. & Townsend, M. U. Tipranavir: the first nonpeptidic protease inhibitor for the treatment of protease resistance. *Clin. Ther.* **29**, 2309–2318 (2007).

71. Croom, K. F., Dhillon, S. & Keam, S. J. Atazanavir: a review of its use in the management of HIV-1 infection. *Drugs* **69**, 1107–1140 (2009).

72. De Clercq, E. Anti-HIV drugs: 25 compounds approved within 25 years after the discovery of HIV. *Int. J. Antimicrob. Agents* **33**, 307–320 (2009).

73. Lalezari, J. P., Ward, D. J., Tomkins, S. A. & Garges, H. P. Preliminary safety and efficacy data of brecanavir, a novel HIV-1 protease inhibitor: 24 week data from study HPR10006. *J. Antimicrob. Chemother.* **60**, 170–174 (2007).

74. Kaldor, S. W. *et al.* Viracept (nelfinavir mesylate, AG1343): a potent, orally bioavailable inhibitor of HIV-1 protease. *J. Med. Chem.* **40**, 3979–3985 (1997).

75. Dierynck, I. *et al.* TMC310911, a novel human immunodeficiency virus type 1 protease inhibitor, shows in vitro an improved resistance profile and higher genetic barrier to resistance compared with current protease inhibitors. *Antimicrob. Agents Chemother.* **55**, 5723–5731 (2011).

76. Izquierdo, L. *et al.* Simeprevir for the treatment of hepatitis C virus infection. *Pharmgenomics. Pers. Med.* **7**, 241–249 (2014).

77. Cotter, T. G. & Jensen, D. M. Glecaprevir/pibrentasvir for the treatment of chronic hepatitis C: design, development, and place in therapy. *Drug Des. Devel. Ther.* **13**, 2565–2577 (2019).

78. Slater, M. J. *et al.* Pyrrolidine-5,5-trans-lactams. 4. Incorporation of a P3/P4 Urea Leads to Potent Intracellular Inhibitors of Hepatitis C Virus NS3/4A Protease. *Org. Lett.* **5**, 4627–4630 (2003).

79. Gentile, I. *et al.* Asunaprevir, a protease inhibitor for the treatment of hepatitis C infection. *Ther Clin Risk Manag.* **10**, 493–504 (2014).

80. Forestier, N. & Zeuzem, S. Telaprevir for the treatment of hepatitis C. *Expert Opin. Pharmacother.* **13**, 593–606 (2012).

81. Treitel, M. *et al.* Single-dose pharmacokinetics of boceprevir in subjects with impaired hepatic or renal function. *Clin. Pharmacokinet.* **51**, 619–628 (2012).

82. Klibanov, O. M., Gale, S. E. & Santevecchi, B. Ombitasvir/paritaprevir/ritonavir and dasabuvir tablets for hepatitis C virus genotype 1 infection. *Ann. Pharmacother.* **49**, 566–581 (2015).

83. Sulejmani, N. & Jafri, S.-M. Grazoprevir/elbasvir for the treatment of adults with chronic hepatitis C: a short review on the clinical evidence and place in therapy. *Hepat. Med.* **10**, 33–42 (2018).

84. Bourlière, M. *et al.* Sofosbuvir, Velpatasvir, and Voxilaprevir for Previously Treated HCV Infection. *N. Engl. J. Med.* **376**, 2134–2146 (2017).

85. Thomson, J. A. & Perni, R. B. Hepatitis C virus NS3-4A protease inhibitors: countering viral subversion in vitro and showing promise in the clinic. *Curr. Opin. Drug Discov. Devel.* **9**, 606–617 (2006).

86. Miao, M., Jing, X., De Clercq, E. & Li, G. Danoprevir for the Treatment of Hepatitis C Virus Infection: Design, Development, and Place in Therapy. *Drug Des. Devel. Ther.* **14**, 2759–2774 (2020).

87. Eltahla, A. A., Tay, E., Douglas, M. W. & White, P. A. Cross-genotypic examination of hepatitis C virus polymerase inhibitors reveals a novel mechanism of action for thumb binders. *Antimicrob. Agents Chemother.* **58**, 7215–7224 (2014).

88. Sun, L. *et al.* Small-molecule inhibition of Aurora kinases triggers spindle checkpoint-independent apoptosis in cancer cells. *Biochem. Pharmacol.* **75**, 1027–1034 (2008).

89. Heinzlmeir, S. *et al.* Chemical Proteomics and Structural Biology Define EPHA2 Inhibition by Clinical Kinase Drugs. *ACS Chem. Biol.* **11**, 3400–3411 (2016).

90. Oslob, J. D. *et al.* Discovery of a potent and selective aurora kinase inhibitor. *Bioorg. Med. Chem. Lett.* **18**, 4880–4884 (2008).

91. Ewart-Toland, A. *et al.* Aurora-A/STK15 T+91A is a general low penetrance cancer susceptibility gene: a meta-analysis of multiple cancer types. *Carcinogenesis* **26**, 1368–1373 (2005).

92. Pacaud, R., Cheray, M., Nadaradjane, A., Vallette, F. M. & Cartron, P.-F. Histone H3 phosphorylation in GBM: a new rational to guide the use of kinase inhibitors in anti-GBM therapy. *Theranostics* **5**, 12–22 (2015).

93. Fancelli, D. *et al.* 1,4,5,6-tetrahydropyrrolo[3,4-c]pyrazoles: identification of a potent Aurora kinase inhibitor with a favorable antitumor kinase inhibition profile. *J. Med. Chem.* **49**, 7247–7251 (2006).

94. Rawson, T. E. *et al.* A pentacyclic aurora kinase inhibitor (AKI-001) with high in vivo potency and oral bioavailability. *J. Med. Chem.* **51**, 4465–4475 (2008).

95. Oslob, J. D. *et al.* Crystal structure of mouse Aurora A (Asn186->Gly, Lys240->Arg, Met302->Leu) in complex with 1-{5-[2-(1-methyl-1H-pyrazolo[4,3-d]pyrimidin-7-ylamino)-ethyl]-thiazol-2-yl}-3-(3-trifluoromethyl-phenyl)-urea. https://www.rcsb.org/structure/3d2i doi:10.2210/pdb3D2I/pdb.

96. Tari, L. W. *et al.* Structural basis for the inhibition of Aurora A kinase by a novel class of high affinity disubstituted pyrimidine inhibitors. *Bioorg. Med. Chem. Lett.* **17**, 688–691 (2007).

97. Cancilla, M. T. *et al.* Discovery of an Aurora kinase inhibitor through site-specific dynamic combinatorial chemistry. *Bioorg. Med. Chem. Lett.* **18**, 3978–3981 (2008).

98. Howard, S. *et al.* Fragment-based discovery of the pyrazol-4-yl urea (AT9283), a multitargeted kinase inhibitor with potent aurora kinase activity. *J. Med. Chem.* **52**, 379–388 (2009).

99. Baluom, M., Grossbard, E. B., Mant, T. & Lau, D. T. W. Pharmacokinetics of fostamatinib, a spleen tyrosine kinase (SYK) inhibitor, in healthy human subjects following single and multiple oral dosing in three phase I studies. *Br. J. Clin. Pharmacol.* **76**, 78–88 (2013).

100. Manfredi, M. G. *et al.* Antitumor activity of MLN8054, an orally active small-molecule inhibitor of Aurora A kinase. *Proc. Natl. Acad. Sci. U. S. A.* **104**, 4106–4111 (2007).

101. Faivre, S., Demetri, G., Sargent, W. & Raymond, E. Molecular basis for sunitinib efficacy and future clinical development. *Nature Reviews Drug Discovery* vol. 6 734–745 (2007).

102. Talpaz, M. *et al.* Dasatinib in imatinib-resistant Philadelphia chromosome-positive leukemias. *N. Engl. J. Med.* **354**, 2531–2541 (2006).

103. Keller-V Amsberg, G. & Brümmendorf, T. H. Novel aspects of therapy with the dual Src and Abl kinase inhibitor bosutinib in chronic myeloid leukemia. *Expert Rev. Anticancer Ther.* **12**, 1121–1127 (2012).

104. Reddy, E. P. & Aggarwal, A. K. The ins and outs of bcr-abl inhibition. *Genes Cancer* **3**, 447–454 (2012).

105. Ahmad, Z., Sharma, S. & Khuller, G. K. Azole antifungals as novel chemotherapeutic agents against murine tuberculosis. *FEMS Microbiol. Lett.* **261**, 181–186 (2006).

106. Byrne, S. T., Denkin, S. M., Gu, P., Nuermberger, E. & Zhang, Y. Activity of ketoconazole against Mycobacterium tuberculosis in vitro and in the mouse model. *J. Med. Microbiol.* **56**, 1047–1051 (2007).

107. Freundlich, J. S. *et al.* Triclosan derivatives: towards potent inhibitors of drug-sensitive and drug-resistant Mycobacterium tuberculosis. *ChemMedChem* **4**, 241–248 (2009).

108. Gold, B. *et al.* Novel Cephalosporins Selectively Active on Nonreplicating Mycobacterium tuberculosis. *J. Med. Chem.* **59**, 6027–6044 (2016).

109. Chambers, H. F. *et al.* Can penicillins and other beta-lactam antibiotics be used to treat tuberculosis? *Antimicrob. Agents Chemother.* **39**, 2620–2624 (1995).

110. Istvan, E. S. *et al.* Validation of isoleucine utilization targets in Plasmodium falciparum. *Proc. Natl. Acad. Sci. U. S. A.* **108**, 1627–1632 (2011).

111. Surolia, N. & Surolia, A. Triclosan offers protection against blood stages of malaria by inhibiting enoyl-ACP reductase of Plasmodium falciparum. *Nat. Med.* **7**, 167–173 (2001).

112. Zhang, Y. & Meshnick, S. R. Inhibition of Plasmodium falciparum dihydropteroate synthetase and growth in vitro by sulfa drugs. *Antimicrob. Agents Chemother.* **35**, 267–271 (1991).

113. Nicholas, R. O., Berry, V., Hunter, P. A. & Kelly, J. A. The antifungal activity of mupirocin. *J. Antimicrob. Chemother.* **43**, 579–582 (1999).

114. Smith, T. F. & Waterman, M. S. Identification of common molecular subsequences. *J. Mol. Biol.* **147**, 195–197 (1981).
